# Integrating Textual Queries with AI-Based Object Detection: A Compositional Prompt-Guided Approach

**DOI:** 10.3390/s25072258

**Published:** 2025-04-03

**Authors:** Silvan Ferreira, Allan Martins, Daniel G. Costa, Ivanovitch Silva

**Affiliations:** 1 Graduate Program in Electrical and Computer Engineering, Federal University of Rio Grande do Norte, Natal 59078-970, Brazil; silvan.junior.051@ufrn.edu.br (S.F.); allan@dee.ufrn.br (A.M.); 2 SYSTEC-ARISE, Faculty of Engineering, University of Porto, 4200-465 Porto, Portugal; danielgcosta@fe.up.pt

**Keywords:** neuro-symbolic AI, prompt-guided object detection, crossmodal reasoning, visual-language alignment, query-driven recognition

## Abstract

While object detection and recognition have been extensively adopted by many applications in decision-making, new algorithms and methodologies have emerged to enhance the automatic identification of target objects. In particular, the rise of deep learning and language models has opened many possibilities in this area, although challenges in contextual query analysis and human interactions persist. This article presents a novel neuro-symbolic object detection framework that aligns object proposals with textual prompts using a deep learning module while enabling logical reasoning through a symbolic module. By integrating deep learning with symbolic reasoning, object detection and scene understanding are considerably enhanced, enabling complex, query-driven interactions. Using a synthetic 3D image dataset, the results demonstrate that this framework effectively generalizes to complex queries, combining simple attribute-based descriptions without explicit training on compound prompts. We present the numerical results and comprehensive discussions, highlighting the potential of our approach for emerging smart applications.

## 1. Introduction

In recent years, automatic object detection and recognition using artificial intelligence (AI) methods has become a critical area of research and application across various domains, including security, healthcare, industrial automation, autonomous vehicles, and robotics [1,2]. With proper sensors, new developments in algorithms to detect a set of target objects has been accelerated, both in accuracy and required computational resources [3,4], enhancing the capabilities for decision-making. In particular, the ability of AI models to accurately identify and classify objects in images or video streams has significantly improved, while increasing computational efficiency has allowed the embedding of such an algorithm in resource-constrained systems [5]. The resulting scenario has fostered the adoption of innovative solutions, although some important challenges still remain.

Image recognition and object detection have benefited from a series of innovations in the area of artificial intelligence, notably considering the deep learning models such as Convolutional Neural Networks (CNNs) and Transformers [6,7]. However, while excelling at identifying “what” is in an image, these models often fall short when it comes to understanding the “how” and “why”. The latter are very important when identifying the intricate relationships between objects, their attributes, and the broader context of a scene, which are critical in emerging smart systems in areas like smart cities and Industry 4.0 [8,9]. Since such domains would benefit from a framework that perceives objects and intelligently reasons about them, the adoption of new mechanisms becomes relevant in this broad scenario.

In fact, traditional object detection frameworks have only focused on particular aspects of localizing and classifying objects, lacking the capability for context-aware interpretation and logical interaction with the detected entities. While recent advances in crossmodal reasoning, particularly in Visual Question Answering (VQA) [10,11,12], have attempted to bridge the gap between visual perception and language understanding, these models often struggle with queries that demand complex logical inferences or relational reasoning, relying heavily on learned statistical correlations rather than explicit symbolic logic. As a result, they provide limited insights into the reasoning process, hindering their explainability and trustworthiness.

Recently, neuro-symbolic AI has been promoted as a way to support new interactions and reasoning within object detection solutions [13]. In short, neuro-symbolic AI combines neural networks with logic-based symbolic reasoning, combining the strengths of deep learning’s perceptual prowess with the structured reasoning capabilities of symbolic logic [14,15,16]. Such a hybrid approach promises AI systems that can perceive and identify objects and reason about their relationships, answer complex queries, and provide transparent explanations for their decisions.

Aiming to improve object detection in multiple scenarios, this article addresses the problem of context-aware, crossmodal neuro-symbolic object detection. By integrating the processing of textual queries with AI-based object recognition, a new framework is proposed to integrate a deep neural network for object detection guided by textual prompts with a symbolic reasoning module that supports complex, query-driven interactions. More specifically, we propose an end-to-end neural architecture that combines a Region Proposal Network (RPN) with a Transformer-based model to achieve precise object detection aligned with user-defined textual prompts.

Our proposed framework introduces a custom query language that facilitates complex interactions with the detected objects through logical and ontological reasoning. The symbolic module interprets alignment scores from the neural module, supporting probabilistic, fuzzy, and Boolean interpretations to handle the inherent uncertainties of crossmodal recognition. To the best of our knowledge, this integrated framework has not been proposed before.

In summary, our contributions are threefold, described as follows:

- A novel framework based on a neural architecture that effectively fuses visual and linguistic information, enabling precise object detection guided by textual prompts;
- A new query language specifically designed for interacting with detected objects, enabling complex logical and ontological reasoning over object attributes and relationships;
- A new synthetic dataset comprising 3D objects with diverse attributes and natural language captions, designed to facilitate research in crossmodal object detection and visual reasoning.

By integrating perceptual and reasoning capabilities, our framework empowers systems to perform contextually aware interactions with objects in complex scenes, demonstrating the significant potential of neuro-symbolic AI to advance scene understanding and object detection.

The remainder of this paper is organized as follows. Section 2 reviews related works, highlighting their strengths and limitations. Section 3 introduces our proposed neuro-symbolic architecture, detailing the neural and symbolic components. Then, Section 4 describes the annotated dataset and experimental setup. The results are presented in Section 5, followed by a discussion of the qualitative insights. Finally, the conclusions and references are presented.

## 2. Related Works

The literature has presented valuable contributions to several related subjects in recent years, influencing our investigation in multiple ways. This section presents some of the most relevant related works, highlighting how they relate to our proposed framework.

Deep learning has transformed object detection and scene understanding, with CNN-based pipelines and region-based frameworks achieving remarkable perceptual accuracy. Models like Faster R-CNN [17], YOLO [18], and SSD [19] laid the groundwork for high-performance object detectors, while Mask R-CNN [20] incorporated instance segmentation for richer scene representations. Building upon these, Oriented R-CNN [21] introduced a two-stage detector with an oriented Region Proposal Network (RPN) that efficiently generates high-quality oriented proposals, enhancing the detection accuracy and speed. Further advancing the field, Mutual-Assistance Learning for Object Detection [22] proposed a robust one-stage detector, MADet, which employs mutual-assistance regression to improve detection performance by leveraging the competition between classification and localization tasks. Although these advances refined visual perception, they did not address more abstract logical or relational reasoning, leaving a gap in the contextual comprehension.

Recent advancements in neuro-symbolic AI have furthered the integration of deep learning with symbolic reasoning, enhancing scene understanding and object detection. Khan et al. [23] provide a comprehensive survey on neuro-symbolic visual reasoning, emphasizing the role of scene graphs and common sense knowledge in achieving rich scene representations and intuitive visual reasoning. Similarly, the work of Colelough [24] systematically reviews neuro-symbolic AI projects, identifying key developments and research gaps. Mashayekhi et al. [25] propose novel methods for incorporating structured reasoning into visual domains, showcasing how symbolic constraints can guide neural modules. These works underscore the potential of combining neural networks with symbolic systems to address complex visual reasoning tasks.

In Visual Question Answering (VQA), significant progress has been made to enhance crossmodal understanding. Zhang et al. [26] propose VQACL, a continual learning framework tailored for evolving multimodal data streams to improve cognitive reasoning. Li et al. [27] extensively survey VQA methodologies, highlighting advancements in Generative Adversarial Networks, autoencoders, and attention mechanisms. Furthermore, Xiao et al. [28] introduce an attention-driven VQA model that improves multimodal alignment by leveraging explicit attention mechanisms for visual and textual features. These contributions highlight the potential for enhancing VQA systems with sophisticated attention and continual learning strategies.

VQA systems heavily depend on effective text encoders to interpret and integrate textual information with visual data. Transformer-based architectures have become foundational in this context due to their proficiency in capturing complex language patterns. For instance, the Layout-Aware Transformer (LaTr) [29] enhances scene-text VQA by incorporating spatial cues, improving the model’s ability to process text within images. Additionally, lightweight transformer models like LiT-4-RSVQA [30] have been developed to handle VQA tasks in resource-constrained environments efficiently. Moreover, the mGTE series [31] introduces generalized text encoders capable of managing long-context text representations across multiple languages, further advancing the integration of textual and visual modalities in VQA systems. These developments underscore the critical role of sophisticated text encoders in enhancing the performance and versatility of VQA models.

To bridge the gap in abstract reasoning, neuro-symbolic frameworks combine low-level perception from neural networks with high-level symbolic reasoning. Early work explored integrating neural perception with rule-based systems [14,15], while more recent architectures focus on tasks like compositional question answering and structured scene interpretation. NS-CL [16] demonstrated how coupling neural object recognition with a symbolic program executor could solve challenging VQA queries. Neural Theorem Provers [32], Neural Logic Machines [33], and Logical Neural Networks [34] are further examples that incorporate logical inference directly into the learning process. By encoding rules as symbolic constraints, these models achieve greater transparency, more substantial generalization to unseen queries, and the capacity to handle complex relational structures.

Some approaches, like CLEVRER [35], integrate reasoning with physical dynamics, while KRISP [36] shows the potential for tighter feedback loops between neural and symbolic modules in real-world scenarios. Such architectures highlight that symbolic logic can guide attention, enforce consistency, and enable explainable decision-making beyond end-to-end neural inference.

Nevertheless, establishing a seamless, scalable integration of deep perception and symbolic reasoning remains challenging. Current methods often specialize in narrow domains, face computational complexity issues, or demand extensive domain-specific rule engineering. Our work builds on these foundations by proposing a framework that achieves robust perceptual grounding via neural modules and incorporates a symbolic layer for general logical queries, providing a flexible, interpretable neuro-symbolic approach to context-aware, crossmodal scene understanding.

Finally, a comparison of the strengths and limitations of various neuro-symbolic approaches is summarized in Table 1.

## 3. Materials and Methods

**Figure 1:**
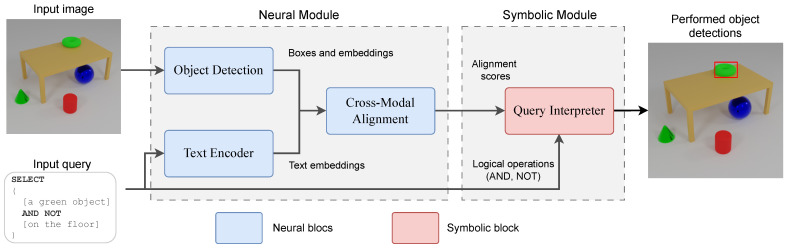
Overview of the proposed framework.

Our proposed neuro-symbolic framework integrates a deep neural network for object detection guided by textual prompts with a symbolic reasoning layer that enables logical query-driven interaction with detected objects. This section details our framework, highlighting the operation of the neural and symbolic modules, as well as the synthetic dataset used for training and evaluation. Figure 1 presents a general schema of our proposed approach.

The neural module is composed of several blocks which are responsible for processing images and text to generate alignment scores between object and textual prompts. The processing blocks within the symbolic module interpret these scores through a pipeline of tokenization, parse into a structured query (AST), perform semantic analysis into an executable query plan, and evaluate to produce the final result. This integration enables context-aware reasoning and crossmodal object detection.

The next subsections describe the characteristics of each processing block that composes our framework.

### 3.1. The Neural Module

The proposed neural module is a fully AI-based component that integrates deep learning techniques to process visual and textual information. The neural module is responsible for detecting objects in images and aligning them with user-defined textual prompts. It consists of four processing blocks: a Region Proposal Network (RPN), an Object Proposal Encoder, a text encoder, and a Proposal-Text Alignment Module.

The core architecture of the neural module follows a structured processing pipeline. First, a RPN generates candidate regions likely to contain objects. These regions are processed via RoI (Region of Interest) pooling to ensure uniform feature dimensions, after which the features are passed through a multi-layer perceptron (MLP) to extract visual embeddings. In parallel, a Transformer-based encoder processes natural language prompts to produce corresponding text embeddings. These two embeddings are subsequently aligned through a shared latent space using a learnable projection, enabling crossmodal comparison between visual and textual modalities.

#### 3.1.1. Region Proposal Network

**Figure 2:**
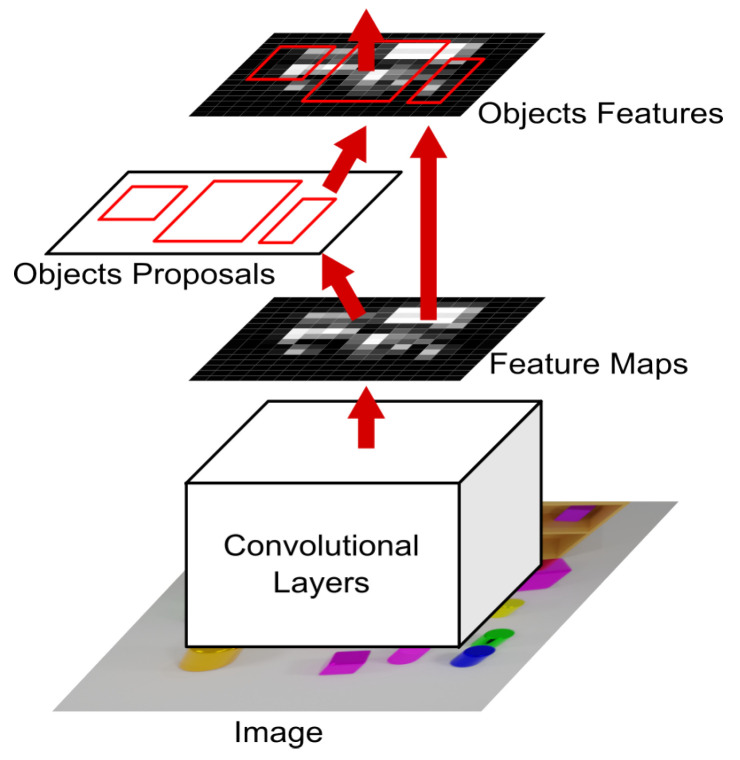
An overview of the Faster R-CNN pipeline, illustrating how the Region Proposal Network (RPN) block generates object proposals, which are then refined and classified to produce final detections.

The Region Proposal Network (RPN) block generates a set of object proposals from an input image. Given an image $I\in R^{H\times W\times 3}$, the RPN processes it through a convolutional feature extractor, producing feature maps of size $H^{\prime }\times W^{\prime }\times C$. Anchors of various scales and aspect ratios are placed over the feature maps to generate *K* region proposals $\left\{R_{1},R_{2},\ldots ,R_{K}\right\}$. Figure 2 further depicts this process.

For each anchor $R_{i}$, the RPN predicts an objectness score $c_{i}$ and bounding box regression parameters $t_{i}=\left(t_{x_{i}},t_{y_{i}},t_{w_{i}},t_{h_{i}}\right)$, where

(1) $$\begin{array}{cccc} t_{x_{i}} & =\frac{x_{i}-x_{a_{i}}}{w_{a_{i}}}, & t_{y_{i}} & =\frac{y_{i}-y_{a_{i}}}{h_{a_{i}}},\\ t_{w_{i}} & =\ln\left(\frac{w_{i}}{w_{a_{i}}}\right), & t_{h_{i}} & =\ln\left(\frac{h_{i}}{h_{a_{i}}}\right), \end{array}$$

with $\left(x_{a_{i}},y_{a_{i}},w_{a_{i}},h_{a_{i}}\right)$ representing the anchor’s center coordinates, width, and height.

The RPN is trained using a multi-task loss function:

(2) $$L_{\mathrm{RPN}}=\frac{1}{N_{\mathrm{cls}}}\sum_{i}L_{\mathrm{cls}}\left(c_{i},c_{i}^{*}\right)+\lambda \frac{1}{N_{\mathrm{reg}}}\sum_{i}c_{i}^{*}L_{\mathrm{reg}}\left(t_{i},t_{i}^{*}\right),$$

where $c_{i}^{*}$ is the ground-truth label (1 for positive anchors, 0 for negative), $t_{i}^{*}$ are the ground-truth bounding box regression targets, $L_{\mathrm{cls}}$ is the binary cross-entropy loss, $L_{\mathrm{reg}}$ is the smooth $L_{1}$ loss, and λ is a balancing hyperparameter.

After training, the RPN outputs a set of refined object proposals, which are then subjected to non-maximum suppression (NMS) to eliminate redundant proposals, resulting in a set $R=\{R_{1},R_{2},\ldots ,R_{N}\}$ of *N* proposals.

#### 3.1.2. Object Proposal Encoder

The Object Proposal Encoder block maps each proposal $R_{i}$ to a fixed-size embedding $v_{i}\in R^{d_{v}}$, where $d_{v}=256$. It is designed to capture both spatial and semantic information for accurate alignment with textual descriptions. For each $R_{i}$, RoI pooling [37] extracts a 7×7×256 feature map $F_{i}$ from the RPN outputs, ensuring uniform representation regardless of the proposal’s original size. Next, $F_{i}$ is passed through three 3×3 convolutional layers (with 256, 512, and 256 filters, each followed by ReLU) to learn higher-level patterns. The transformed features are flattened and fed into two fully connected layers (1024 units with ReLU, then 256 units) to produce the final embedding vector $v_{i}$. We apply L2-normalization:

(3) $$v_{i}=\frac{W_{e}\;\mathrm{vec}\left(F_{i}\right)+b_{e}}{∥W_{e}\;\mathrm{vec}\left(F_{i}\right)+b_{e}{∥}_{2}},$$

where $W_{e}$ and $b_{e}$ are the embedding layer parameters, and vec(·) denotes vectorization. This normalization ensures the embeddings lie on a hypersphere, facilitating alignment via cosine similarity.

#### 3.1.3. Text Encoder

The text encoder block maps each textual prompt $T_{j}$ to a fixed-size embedding $u_{j}\in R^{d_{u}}$. A Transformer-based architecture [7] captures the semantic meaning of the prompt. First, $T_{j}$ is tokenized into words or subword units and mapped to embedding vectors; positional encodings are then added to preserve token order. Multiple self-attention layers follow, capturing contextual relationships between tokens. The output is aggregated (e.g., via mean or max pooling) and passed through fully connected layers to produce the embedding $u_{j}$, which is then L2-normalized:

(4) $$u_{j}=\frac{W_{t}\;\mathrm{Aggregate}\left(H_{j}\right)+b_{t}}{{∥W_{t}\;\mathrm{Aggregate}\left(H_{j}\right)+b_{t}∥}_{2}},$$

where $H_{j}$ represents the outputs from the self-attention layers. This approach allows the encoder to handle complex prompts by capturing the relationships between words, enabling meaningful comparisons with the object proposal embeddings.

#### 3.1.4. Proposal-Text Alignment Module

**Figure 3:**
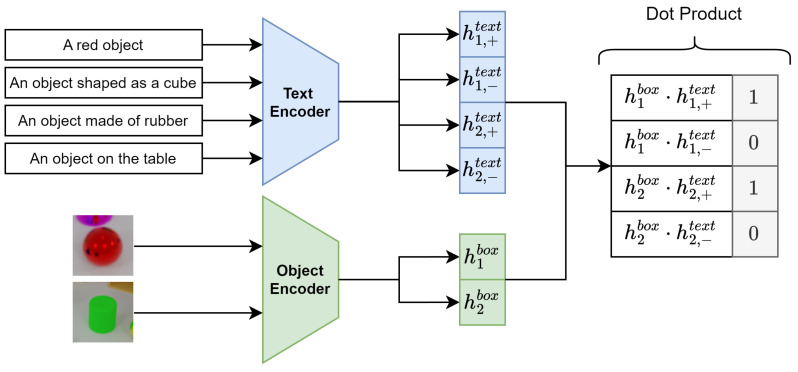
The Proposal-Text Alignment block, demonstrating how object proposal embeddings and text embeddings are projected into a common latent space and aligned via cosine similarity.

As shown in Figure 3, this processing block computes alignment scores between object proposals and textual prompts by projecting their embeddings into a common latent space and measuring their similarity.

Given the object proposal embeddings ${\left\{v_{i}\right\}}_{i=1}^{N}\subset R^{d_{v}}$ from the Object Proposal Encoder and the text embeddings ${\left\{u_{j}\right\}}_{j=1}^{M}\subset R^{d_{u}}$ from the Text Encoder, the alignment proceeds as follows. First, each embedding is projected into a shared latent space of dimension $d_{h}$ via learnable linear transformations:

(5) $$\begin{array}{cc} h_{i}^{\mathrm{box}} & =W_{b}v_{i}+b_{b},\;W_{b}\in R^{d_{h}\times d_{v}},\;b_{b}\in R^{d_{h}}, \end{array}$$

(6) $$\begin{array}{cc} h_{j}^{\mathrm{text}} & =W_{t}u_{j}+b_{t},\;W_{t}\in R^{d_{h}\times d_{u}},\;b_{t}\in R^{d_{h}}. \end{array}$$

Next, the projected embeddings are normalized:

(7) $$\begin{array}{cc} {\hat{h}}_{i}^{\mathrm{box}} & =\frac{h_{i}^{\mathrm{box}}}{∥h_{i}^{\mathrm{box}}{∥}_{2}}, \end{array}$$

(8) $$\begin{array}{cc} {\hat{h}}_{j}^{\mathrm{text}} & =\frac{h_{j}^{\mathrm{text}}}{∥h_{j}^{\mathrm{text}}{∥}_{2}}. \end{array}$$

The alignment score between proposal *i* and prompt *j* is then the cosine similarity of the normalized embeddings:

(9) $$s_{ij}={\hat{h}}_{i}^{\mathrm{box}}\cdot {\hat{h}}_{j}^{\mathrm{text}}=\langle {\hat{h}}_{i}^{\mathrm{box}},{\hat{h}}_{j}^{\mathrm{text}}\rangle .$$

To map $s_{ij}$ from [−1,1] to [0,1], we apply a linear transformation:

(10) $$s_{ij}=\frac{s_{ij}+1}{2}.$$

Finally, the alignment module is trained using a binary cross-entropy loss between the predicted alignment scores and ground-truth labels:

(11) $$L_{\mathrm{align}}=-\frac{1}{NM}\sum_{i=1}^{N}\sum_{j=1}^{M}\left[y_{ij}\log\left(s_{ij}\right)+\left(1-y_{ij}\right)\log\left(1-s_{ij}\right)\right],$$

where the binary label $y_{ij}$ is set to 1 if the ground-truth object represented by proposal $R_{i}$ matches the semantic content of prompt $T_{j}$, based on the dataset labels. Otherwise, it is set to 0.

### 3.2. The Symbolic Module

Using a custom query language, the symbolic module enables logical reasoning and complex interactions with the detected objects. It interprets the alignment scores from the neural module and applies logical operations to execute queries. The module comprises four key stages:

- Lexer (Tokenizer): The lexer processes the raw query string and produces a stream of tokens, each representing the smallest meaningful unit (e.g., PROMPT,OPERATOR, and NUMBER). Whitespace and comments are discarded, allowing subsequent parsing steps to work with well-defined tokens.
- Parser (AST Construction): The parser reads the token stream and constructs an abstract syntax tree (AST) based on predefined grammar rules. For example, [a red object] and [an object on the floor] are transformed into a logical conjunction node with two prompt node children, while expressions like COUNT [a red object] > 5 generate a comparison node linking a COUNT operation to a NUMBER.
- Semantic Analyzer: This stage checks the consistency and data types throughout the AST. It ensures that logical operators apply to Boolean-like or fuzzy/probabilistic scores, arithmetic operators appear only with numeric data, and each prompt node is assigned an uncertainty interpretation (Boolean, fuzzy, or probabilistic).
- Evaluator (Query Execution): Finally, the evaluator executes the AST by fetching alignment scores from the neural module and applying the user-defined operations.

### 3.3. Proposed Query Language

In order to support the operation of our proposed framework, a new query language is defined to support conceptual object detection. The proposed query language supports three primary data types. The NUMBER type represents integers or real numbers, BOOLEAN accepts only true or false, and PROMPT consists of natural language text enclosed in brackets.

The supported operators include arithmetic, logical, and comparison operators. Specifically, ^ is used for exponentiation (precedence level 1); *, /, and % denote multiplication, division, and modulus operations (precedence level 2); + and − perform addition and subtraction (precedence level 3); comparison operators (==, !=, <, <=, >, >=) have a precedence level of 4; logical NOT (not) operates at level 5; logical AND (and) at level 6; logical XOR (xor) at level 7; and logical OR (or) at level 8.

#### 3.3.1. Interpreting Scores

The interpretation of the alignment scores influences how logical operations are computed. Three approaches are considered: probabilistic, fuzzy, and Boolean. In the probabilistic interpretation, scores are treated as probabilities and operations use probability theory (e.g., the product rule for AND and the inclusion–exclusion principle for OR). In the fuzzy interpretation, scores represent degrees of truth with operations defined via fuzzy logic (using the minimum for AND and maximum for OR). In the Boolean interpretation, scores are binarized using a threshold (e.g., θ=0.5) before applying standard Boolean algebra.

#### 3.3.2. Clause Structures

The query language provides clauses for interacting with detected objects, which can be combined with logical operators to form complex queries.

- Select: Identifies entities matching a prompt, returning object indices above a score threshold.
  – How to use:
select [entity selection]
select sort by [attribute] asc/desc limit N
  – *Example:* To select the three biggest green objects:
select [an object with color green]
sort by [a big object] desc limit 3
- Count: Counts the number of entities matching a prompt, returning an integer.
  – How to use:
count [entity selection]
  – *Example:* Number of objects on the floor:
count [an object on the floor]
- All and Any: Checks whether all or any entities satisfy a given condition, returning a Boolean.
  – How to use:
all/any [entity selection]
  – *Example:* Check if all objects are made of metal:
all [an object made of metal]
  – *Example:* Check if at least one object is inside the box:
any [an object inside the box]

### 3.4. Integration and Uncertainty Handling

The interaction between the neural and symbolic modules is achieved through the alignment scores provided by the neural module. The symbolic module leverages these scores as inputs for its logical operations, thereby grounding symbolic reasoning in the perceptual outputs. This integration enables the complex, context-aware analysis of detected objects by combining neural perception with symbolic logic.

To handle uncertainties inherent in crossmodal recognition, the framework supports three interpretations of the alignment scores: probabilistic, fuzzy, and Boolean. The choice of interpretation affects how evidence from multiple conditions is aggregated when executing complex queries.

- Probabilistic Interpretation: Alignment scores $s_{ij}$ are treated as probabilities, representing the likelihood that object proposal $R_{i}$ corresponds to textual prompt $T_{j}$. Logical operations are performed using probabilistic logic.
- Fuzzy Interpretation: Alignment scores are interpreted as degrees of truth. Logical operations are defined according to fuzzy set theory—for example, the AND operation is implemented as the minimum of the scores, while the OR operation is implemented as the maximum.
- Boolean Interpretation: Scores are first binarized using a threshold θ (typically θ=0.5), after which standard Boolean algebra applies.

This flexibility in score interpretation enhances system adaptability and enables experimentation with different reasoning paradigms. The integration ensures that uncertainties at the perceptual level are propagated and incorporated during the reasoning process, yielding robust and interpretable results, even in the presence of noise or ambiguity.

**Figure 4:**
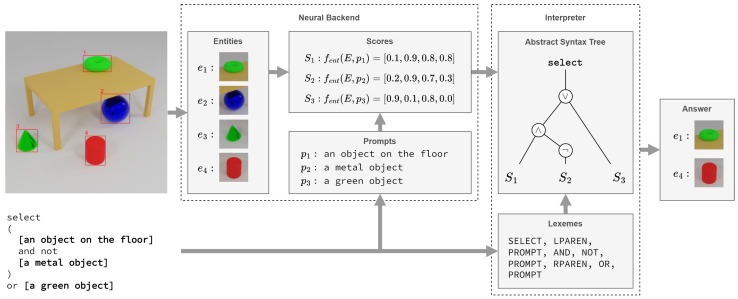
The neural module (**left**) takes input images and textual prompts, generating object proposals and producing alignment scores between visual features and text. The symbolic module (**right**) uses these scores to execute logical queries, enabling context-aware, explainable reasoning over detected objects.

Based on the defined query language and modules, users can search for objects with different characteristics. Figure 4 presents a query example. By fusing perception with symbolic inference, the system can isolate specific entities—such as selecting “an object on the floor” but “not a metal object’ and provide transparent answers, even when multiple attributes must be satisfied or ruled out. 

## 4. Dataset

**Figure 5:**
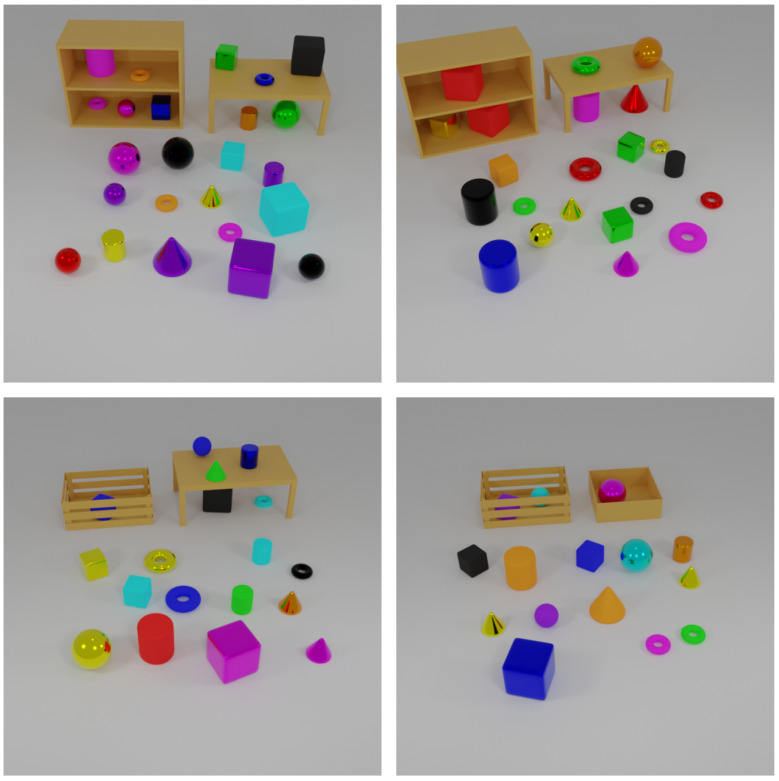
Examples from the AOVR-Dataset. Each image depicts 3D objects exhibiting diverse shapes, colors, materials, and sizes in different container settings. Bounding box annotations and natural language descriptions accompany each object.

To train and evaluate our framework, we construct the Annotated Objects for Visual Reasoning Dataset (AOVR-Dataset), a synthetic 3D dataset designed to facilitate research in visual reasoning and object detection. The dataset comprises 3D objects arranged in various container settings, with each object annotated using precise bounding boxes and natural language descriptions. Examples of the generated 3D scenes with annotated objects from the AOVR-Dataset are shown in Figure 5.

The dataset is publicly available at https://data.mendeley.com/datasets/bn5cbjts6j/1 (accessed on 24 February 2025).

### 4.1. Dataset Description

The AOVR-Dataset was created to support tasks in visual reasoning, object detection, and natural language understanding. It encompasses a wide variety of objects with multiple attributes. The objects come in shapes such as cylinder, cube, torus, cone, and sphere; display colors including blue, magenta, black, red, orange, purple, green, yellow, and cyan; are made from materials like metal and rubber; and are classified as small or big. Additionally, objects are placed in various containers including floor, shelf bottom, shelf top, table top, table under, crate, box, shelf, and table. Each object is annotated with precise bounding boxes for localization and natural language descriptions—captions are generated using a Large Language Model (LLM) to provide context-rich details.

### 4.2. Dataset Generation

Three-dimensional models are created using Blender (version 3.4), a professional open-source 3D graphics software, ensuring diverse spatial configurations and contexts. Objects are rendered in multiple scenes to capture varied arrangements. Natural language descriptions are generated by an LLM to incorporate diverse expressions and synonyms for each attribute, thereby enhancing the dataset’s applicability in training models to associate visual features with varied linguistic expressions.

For each image, a corresponding JSON file with the same name is generated, providing a structured annotation of all objects in the scene. Each object entry includes visual attributes, spatial location in 3D space, container information, and a 2D bounding box for image-level localization. An example of such a JSON file is shown in Listing 1.

**Listing 1.** Example of JSON annotation for a single image.

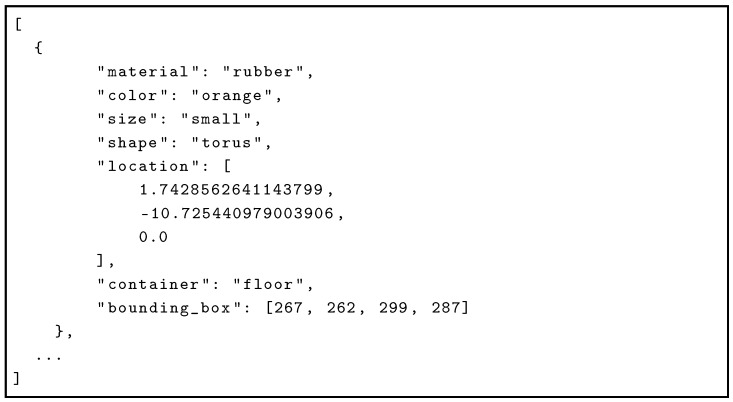



The process of generating captions for attributes involves systematically querying a LLM to create diverse linguistic descriptions associated with specific visual properties. For each attribute–value pair, e.g., color: orange, the LLM is prompted explicitly to generate multiple unique, lowercase sentences describing that property, avoiding redundancy and extraneous information. Specifically, using the GPT-4o model from OpenAI, each attribute is expanded into a set of 100 distinct descriptive phrases. The prompt used to generate sentences is shown in Listing 2.

**Listing 2.** Prompt used to generate captions for each attribute.

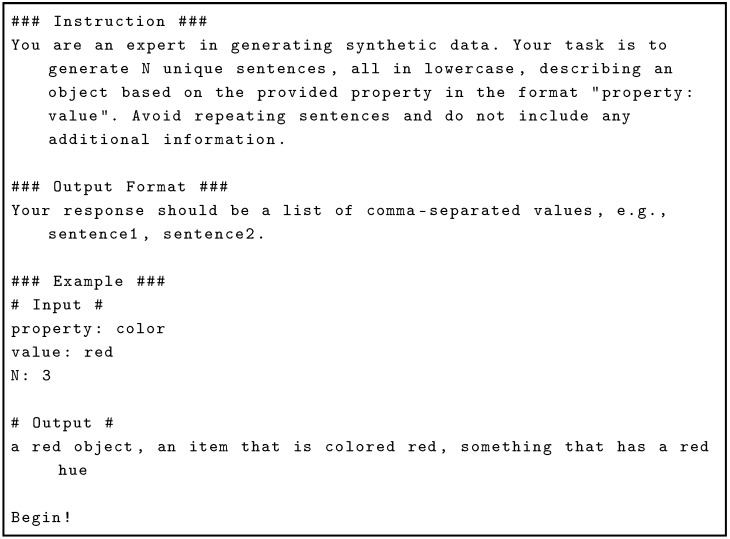



### 4.3. Annotations Examples

Table 2 presents examples of natural language descriptions for different object attributes.

## 5. Results

In this section, we evaluate the performance of our proposed neuro-symbolic framework on the AOVR-Dataset. Our experiments aim to demonstrate the framework’s effectiveness in object detection, alignment with textual prompts, and handling complex queries via the symbolic module. We present the experimental setup, including dataset details and implementation specifics, followed by results and analysis.

### 5.1. Preparing the Dataset

The AOVR-Dataset comprises 10,000 examples, partitioned into a training set of 8000 and a validation set of 2000. Each example consists of an image with annotated objects and associated textual prompts. The dataset includes various objects, attributes, and scenes to ensure effective generalization. The validation set contains novel combinations of attributes to assess the model’s ability to handle new scenarios.

### 5.2. Implementation Details

**Figure 6:**
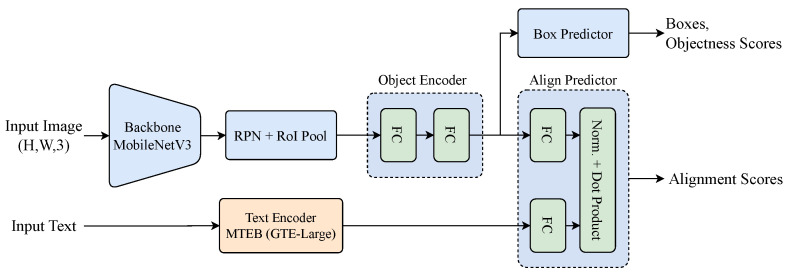
Overview of the neural module.

Our neural network is implemented in PyTorch (version 2.5.1)  [38]. The RPN uses a lightweight MobileNetV3 backbone [39], pretrained on ImageNet [40], chosen for its efficiency and strong performance in object detection. The Object Proposal Encoder is a two-layer MLP with 1024 neurons per layer. For text, we use the large version of the Transformer-based mGTE encoder [41], trained on large-scale multilingual data with 1024-dimensional embeddings, enabling rich semantic understanding. Both visual and textual embeddings are projected into a shared 64-dimensional space by the Proposal-Text Alignment Module, which computes alignment scores via element-wise multiplication and summation. All components are trained end-to-end for joint optimization. Figure 6 presents the implemented neural module architecture.

### 5.3. Training Procedure

**Figure 7:**
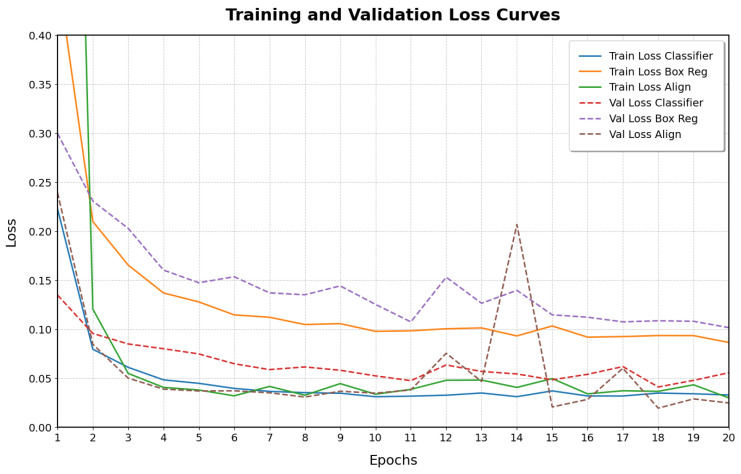
Training and validation loss curves during model training.

Model training is conducted using the AdamW optimizer [42] with an initial learning rate of $1\times 10^{-3}$. We employ a learning rate scheduler that dynamically reduces the learning rate based on the validation loss, using a “reduce on plateau” strategy. Specifically, the learning rate is reduced when the validation loss ceases to improve, ensuring optimal convergence during training. The model is trained for 20 epochs with a batch size of 16. Both training and inference are performed on an NVIDIA GeForce GTX 3060 GPU equipped with 12 GB of VRAM. The model is trained by minimizing the combined loss function:

(12) $$L=L_{\mathrm{RPN}}+L_{\mathrm{align}},$$

where $L_{\mathrm{RPN}}$ encourages accurate object proposal generation, and $L_{\mathrm{align}}$ ensures correct association between visual features and textual descriptions. Figure 7 shows the training and validation loss curves over the 20 epochs. Both training and validation losses decrease and converge, indicating that the model learns effectively without significant overfitting.

#### Evaluation Metrics

We evaluate our framework using the following metrics:

- Mean Average Precision (mAP): Assesses object detection performance, calculated at Intersection over Union (IoU) thresholds of 0.5 and 0.75.
- Average Intersection over Union (IoU): Measures the average overlap between predicted bounding boxes and ground truth.
- Alignment Accuracy: Measures the accuracy of aligning object proposals with textual prompts, expressed as the percentage of correct alignments.
- Query Execution Accuracy: Evaluates the correctness of answers produced by the symbolic module when executing queries, compared against ground-truth answers.

### 5.4. Object Detection Performance

To assess the baseline object detection capabilities of the neural module without considering alignment with textual prompts, the performed evaluation aims to determine the model’s effectiveness in accurately localizing objects within images.

We evaluate the object detection performance of the neural module on the validation set of the AOVR-Dataset. The evaluation procedure involves the following steps:

1. Model Inference: The model processes each image in the validation set, generating predicted bounding boxes and associated objectness scores for detected objects.
2. Performance Metrics: We compute the mean Average Precision (mAP) at Intersection over Union (IoU) thresholds of 0.5 and 0.75. The mAP metric provides a comprehensive evaluation by considering the precision and recall across different confidence levels. Additionally, we calculate the average IoU across all detected objects to assess the precision of the bounding box localization.
3. Evaluation Protocol: The mAP is calculated following the standard protocol used in object detection benchmarks, where precision and recall are evaluated at varying confidence thresholds, and the average is taken over all thresholds.

The model achieves an mAP of 99.48% at IoU = 0.5 and 98.01% at IoU = 0.75, with an average IoU of 93.39%.

The results indicate that the neural module achieves high detection accuracy on the AOVR-Dataset. The mAP of 99.48% at an IoU threshold of 0.5 demonstrates the model’s effectiveness in correctly identifying and localizing objects within the images.

At a stricter IoU threshold of 0.75, the mAP remains high at 98.01%, suggesting that the predicted bounding boxes closely match the ground truth annotations even under more stringent overlap criteria. The average IoU of 93.39% further confirms the precision of the bounding box predictions.

This high localization accuracy is critical for ensuring that the detected objects correspond accurately to the entities present in the scenes, which is essential for subsequent tasks such as alignment with textual prompts and symbolic reasoning.

### 5.5. Alignment Performance

To evaluate the model’s ability to correctly align object proposals with their corresponding textual prompts, effectively associating visual features with semantic descriptions, this assessment focuses on the model’s capability to distinguish between correct (positive) and incorrect (negative) textual descriptions for each detected object using prompts that refer to a single attribute.

We assess the alignment performance of our model using a controlled experiment designed to test its ability to differentiate between positive and negative captions for each object proposal. The experiment is conducted as follows:

1. Data Preparation: For each object in the validation set, we generate a set of positive captions that accurately describe a single attribute of the object (e.g., [a red object], [a cube-shaped object]). Additionally, we generate negative captions that describe an attribute not present in the object (e.g., [a green object] for a red object). Positive and negative captions are balanced to ensure the equal representation of correct and incorrect prompts. This ensures that the model is tested on its ability to recognize correct descriptions and reject incorrect ones based on single attributes.
2. Feature Extraction: The neural module processes each detected object proposal to extract visual embeddings representing the object’s features, and the text encoder converts both positive and negative captions into textual embeddings.
3. Alignment Score Computation: We compute alignment scores with both their positive and negative captions using the Proposal-Text Alignment Module for each object proposal. A confidence threshold set at 0.5 is applied to the alignment scores to generate binary predictions.
4. Accuracy Calculation: We compare the model’s predictions with the ground truth labels. The alignment accuracy is calculated as the percentage of correct predictions from the total number of caption–object pairs evaluated.

The model achieves an alignment accuracy of 99.35%, indicating that it correctly aligns object proposals with their corresponding positive captions and rejects negative captions with high precision.

The high alignment accuracy indicates that the model effectively associates the visual features with corresponding textual descriptions based on single attributes. This performance suggests the model successfully captures the semantic relationships between objects and language.

### 5.6. Query Execution Accuracy Analysis with Uncertainty Handling Methods

To evaluate the model’s ability to handle complex queries involving multiple object attributes and assess the impact of different uncertainty handling methods in the symbolic module on query execution accuracy, we aim to highlight the advantages of using a symbolic method to combine multiple simple prompts over training the model with complex sentences.

We design an experiment where the model is trained exclusively on simple prompts, each describing a single attribute of an object (e.g., [a red object]). During the evaluation, we test the model’s capacity to handle complex queries constructed by combining multiple simple prompts using logical and operations within the symbolic module. This approach leverages symbolic reasoning to interpret and execute complex queries without requiring the neural model to process complex sentences directly.

The experiment is conducted as follows:

1. Attribute Selection: For each of 1000 images from the test set, randomly select attributes to create queries of varying lengths (*n*), where *n* ranges from 1 to 5.
2. Query Generation: Generate complex queries by combining *n* randomly chosen attributes using logical and operations. For example, a query with n=3 could be the following:
  select [a red object]
  and [a metal object]
  and [an object on the floor]
3. Ground Truth Extraction: Identify the ground-truth bounding boxes of objects that satisfy all selected attributes.
4. Model Inference and Reasoning: Use the neural module to generate object proposals and compute alignment scores for each simple attribute prompt. Employ the symbolic module to execute the complex query under three different uncertainty handling interpretations:
  - Probabilistic Interpretation (PI): Treat alignment scores as probabilities and combine them using probabilistic logic.
  - Fuzzy Interpretation (FI): Interpret scores as degrees of truth in fuzzy logic and combine them accordingly.
  - Boolean Interpretation (BI): Binarize alignment scores using a threshold (e.g., 0.5) and apply standard Boolean logic.
5. Accuracy Computation: Calculate the query execution accuracy by comparing the model’s predicted bounding boxes with the ground truth. A prediction is correct if the Intersection over Union (IoU) exceeds 0.5.

The query execution accuracy for each number of attributes and uncertainty handling method is presented in Table 3.

The results demonstrate that the model effectively handles complex queries by combining simple prompts using the symbolic module. Even though the model is trained only on simple prompts, it achieves reasonable accuracy on complex queries involving multiple attributes.

We observe a decreasing trend in accuracy as the number of attributes in the query increases across all uncertainty handling methods. This decline aligns with theoretical expectations based on the probabilistic combination of independent attribute recognitions.

Assuming that the recognition of each attribute is an independent event with an individual attribute recognition accuracy of approximately 91%, the expected accuracy for queries involving *n* attributes can be approximated by

$$\mathrm{Expected}\;\mathrm{Accuracy}={\left(0.91\right)}^{n}\times 100\%.$$

Comparing the calculated theoretical values with the observed accuracies in Table 3, we find that they closely align, confirming that the decline in accuracy is mainly due to the compounding effect of combining multiple attributes.

Regarding the impact of different uncertainty handling methods, we observe that the differences in accuracy among the probabilistic, fuzzy, and Boolean interpretations are relatively small, generally within 1% to 2% of each other. This suggests that the system is robust to the choice of the uncertainty handling method. Additionally, for queries with a higher number of attributes (n=5), the fuzzy interpretation achieves slightly higher accuracy, possibly because fuzzy logic better handles partial truths in complex combinations.

These observations highlight the effectiveness of using a symbolic module to combine simple prompts, allowing the system to handle complex queries without requiring the neural model to process complex sentences directly. This approach offers the following advantages:

- Scalability: Training the model exclusively on simple prompts reduces the complexity of the training data and the model. It eliminates the need to cover all possible combinations of attributes during training, which would be impractical due to the combinatorial explosion of possible complex sentences.
- Modularity: The symbolic module provides a flexible framework to combine simple concepts into more complex ones. This modularity allows for easier debugging, extension, and maintenance of the system.
- Interpretability: By using a symbolic method to combine attributes, the reasoning process becomes more transparent and interpretable. Each step of the logical combination is explicit, facilitating understanding and trust in the system’s decisions.
- Generalization: The approach allows the model to generalize to unseen combinations of attributes. Since the neural module learns representations for individual attributes, the symbolic module can recombine these to handle novel queries without additional training.

### 5.7. Qualitative Analysis

**Figure 8:**
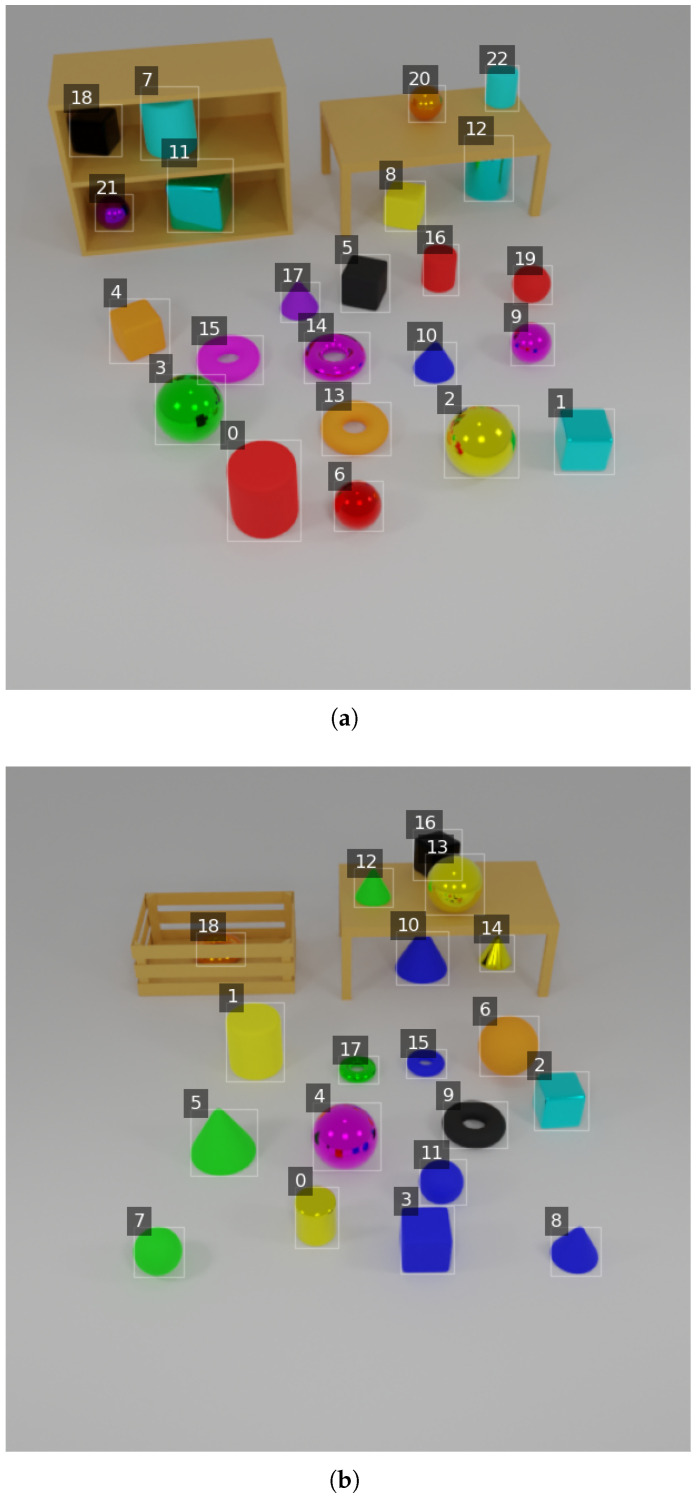
Images annotated with bounding boxes and object indices for qualitative analysis. (**a**) Objects for tests in basic attributes and logical queries. (**b**) Objects for quantitative and comparative queries.

This section demonstrates the interpretability of our neuro-symbolic framework through logical queries executed on selected images from the AOVR-Dataset. Each test evaluates the system’s ability to handle complex reasoning tasks by combining alignment scores from the neural module with logical operations from the symbolic module. Figure 8a,b display images annotated with bounding boxes and object indices (e.g., 1, 2, and 3), followed by the query descriptions and results.

#### 5.7.1. Test 1: Basic Attribute and Logical Queries

Table 4 presents a series of queries involving basic attributes such as color, shape, and spatial location, composed using logical operations like and, or, and not. The first five examples produce correct outputs, demonstrating the framework’s capability to handle simple property-based filtering and basic logical composition.

An interesting observation arises from the query select [a green object or a donut], which returns the expected result despite the likely absence of explicit logical disjunction modeling in the encoder. This behavior can be attributed to the fact that the embedding of the entire phrase effectively captures both “green object” and “donut” as distinct visual–linguistic patterns. Since these attributes are disjoint in the dataset, the model retrieves objects matching either one, even though the logical “or” is not explicitly parsed.

Conversely, the query select [a red colored cylinder] does not return the correct result, instead producing a superset of relevant and irrelevant objects. This discrepancy is likely due to the text encoder’s limited capacity to enforce logical conjunction. In practice, the encoder does not interpret conjunctions such as “and” or composite phrases as strict intersections of properties. Rather, it embeds the entire phrase into a joint vector representation, where multiple attributes are blended rather than logically composed. As a result, the model tends to retrieve objects that exhibit partial matches to the combined description.

These results suggest that the system relies on the emergent alignment between textual and visual embeddings, rather than explicit logical parsing. In cases where precise attribute combination is required, the absence of symbolic composition limits the model’s ability to produce exact matches.

#### 5.7.2. Test 2: Quantitative and Comparative Queries

Test 2 focuses on quantitative and comparative reasoning, including object counting, ratio calculations, and threshold comparisons. The first six queries in Table 5 show consistent results, indicating that the system can reliably count objects based on attributes, combine conditions, and compare numerical values.

The query count [a metal object] correctly returns the number of metallic items, and the comparison count [a metal object] > 5 is also evaluated correctly. The total object count and the ratio computation count [a sphere]/count [*] both match the ground truth, showing that the system handles basic arithmetic over counts. Also, the query count ([a blue object] and [on the floor]) confirms that compound filters are working numerically, and the model is able to compare quantities across distinct categories as seen in the query comparing blue objects on the floor to green ones.

However, the last two rows in Table 5 highlight the limitations. The count of pink objects is off by one, and the model incorrectly identifies six black spheres, while none exist in the ground truth. These errors suggest the model is susceptible to blending or misinterpreting multi-attribute combinations. This shows a similar behavior observed in Test 1: the model tends to match semantically close but incorrect instances when multiple conditions are tightly bound in a single phrase.

### 5.8. Threats to Validity

An initial limitation of this study is the lack of an experiment comparing the model’s performance when trained without complex prompts. While our approach demonstrates the effectiveness of compositional prompts, we did not test whether training the model with only simpler prompts would lead to significantly worse results. Such an analysis could provide a clearer understanding of the direct contribution of complex prompts and whether simpler training strategies might be sufficient.

Another potential threat to validity is the absence of direct comparisons with other existing models. Despite our efforts, we did not find a directly comparable approach in the literature that addresses the same problem. Because of this, while our results indicate that our method is effective, we cannot fully assess how it performs relative to alternative solutions. Future work could explore similar methodologies or adapt related models to serve as benchmarks.

Lastly, we did not test the model on multiple datasets due to the lack of available datasets designed for this specific task. This limitation makes it difficult to determine how well the model would generalize to different data distributions. Although the results are promising within the chosen dataset, further validation on diverse datasets would strengthen the findings and provide a broader evaluation of the approach’s robustness. Future research could focus on curating new datasets or adapting existing ones to enable more comprehensive testing.

## 6. Conclusions

This paper introduced a neuro-symbolic framework that integrates deep learning and symbolic reasoning to improve object detection and scene understanding. The proposed system combines a neural module for object detection and visual–textual alignment with a symbolic module for logical reasoning and query execution.

The neural module, based on a Region Proposal Network and Transformer-based encoders, achieved strong performance in detecting objects and associating them with textual prompts. The model reached a mean average precision of 99.48% at IoU = 0.5 and an alignment accuracy of 99.35%. The symbolic module, supported by a custom query language and flexible uncertainty handling, enabled precise and interpretable reasoning over detected objects and their attributes.

Experimental results demonstrated that complex, compositional queries could be executed without requiring the neural model to be trained on compound sentences. The accuracy for multi-attribute queries followed expected probabilistic trends, confirming the effectiveness of combining simple concepts symbolically. Qualitative analyses highlighted the interpretability of the framework, providing transparent outputs through logical composition.

In addition, the AOVR-Dataset, developed to support this study, offers a structured and diverse benchmark for crossmodal reasoning and object detection. It contributes a valuable resource for future research in neuro-symbolic AI.

Future work includes extending the reasoning capabilities to support temporal and spatial logic, integrating common-sense knowledge, and applying the framework in real-world tasks such as robotics and interactive environments.

## Figures and Tables

**Table 1:** Pros and cons of existing neuro-symbolic approaches.

Approach	Pros	Cons
Early Hybrid Models	Straightforward rules Clear symbolic logic	Limited scalability Rigid rule design
Neural Theorem Provers	End-to-end training Complex inferences	High compute cost Needs large data
NS-CL-like Methods	Good compositionality More interpretability	Domain restrictions Fragile logical forms
Transformer-based VQA	Strong perception Good language alignment	Weak deep reasoning Heavily data driven
Our Framework	Flexible neural-symbolic integration Handles uncertainties Custom query language	Requires alignment training Dataset design effort

**Table 2:** Examples of natural language descriptions for object attributes.

Attribute	Descriptions
Shape: Cylinder	- a cylindrical object - an item shaped like a cylinder - something that has a cylindrical form
Color: Red	- a red object - an item that is colored red - something that has a red hue
Material: Metal	- a metal object - an item made of metal - something that is constructed from metal
Size: Big	- a big object - an item that is large in size - something that occupies a lot of space
Container: Shelf Top	- a container placed on the top of a shelf - an item located on the shelf’s upper surface - a shelf top container

**Table 3:** Query execution accuracy.

Number of Attributes (*n*)	Probabilistic (%)	Fuzzy (%)	Boolean (%)
1	91.06	90.49	91.30
2	82.62	83.83	82.87
3	74.88	74.69	75.27
4	68.44	67.65	69.05
5	62.52	64.16	62.85

**Table 4:** Examples of attribute-based and logical queries, including logical combinations, along with their outputs and ground truth.

Description	Query	Output	Ground Truth
Selects red, spherical, metallic objects	select [a red object] and [an object shaped like a ball] and [a metallic piece]	Indices([6])	Indices([6])
Selects purple objects not in the shelf	select [a purple object] and not [an object in the shelf]	Indices([17])	Indices([17])
Selects the largest red cylinder	select [a cylinder] and [a red object] sort_by [a big object] limit 1 desc	Indices([0])	Indices([0])
Selects black objects not on the floor	select [a black object] and not [an object on the floor]	Indices([18])	Indices([18])
Checks if there is at least one purple cone-shaped object	any [a purple object] and [an object shaped like a cone]	Boolean(True)	Boolean(True)
Selects green objects or objects shaped like donuts	select [a green object or a donut]	Indices([15, 3, 13, 14])	Indices([15, 3, 13, 14])
Selects an object on the table	select [an object on the table]	Indices([20])	Indices([20, 22])
Selects a red cylinder	select [a red colored cylinder]	Indices([12, 7, 19, 6, 22, 0, 16])	Indices([0])

**Table 5:** Examples of quantitative and comparative queries, covering counts, ratios, and logical evaluations over object sets.

Description	Query	Output	Ground Truth
Counts objects made of metal	count [a metal object]	Number(8)	Number(8)
Checks if metal objects exceed 5	count [a metal object] > 5	Boolean(True)	Boolean(True)
Counts all objects	count [*]	Number(19)	Number(19)
Calculates the ratio of spheres to total objects	count [a sphere]/count [*]	Number(0.263157)	Number(0.263157)
Counts blue objects on the floor	count ([a blue object] and [on the floor])	Number(5)	Number(5)
Checks if blue objects on the floor exceed green objects	count ([a blue object] and [on the floor]) > count [a green thing]	Boolean(True)	Boolean(True)
Counts pink objects	count [a pink object]	Number(2)	Number(1)
Counts black spheres	count [a black sphere]	Number(6)	Number(0)

## Data Availability

The original data presented in the study are openly available in Mendeley Data at https://doi.org/10.17632/bn5cbjts6j.1.
